# Psycho‐social factors associated with disagreement between prospective and retrospective measures of childhood maltreatment

**DOI:** 10.1111/jcpp.70129

**Published:** 2026-01-31

**Authors:** Oonagh Coleman, Jessie R. Baldwin, Louise Arseneault, Helen L. Fisher, Terrie E. Moffitt, Andrea Danese

**Affiliations:** ^1^ Social, Genetic and Developmental Psychiatry Centre, Institute of Psychiatry, Psychology & Neuroscience King's College London London UK; ^2^ Department of Clinical, Educational and Health Psychology, Division of Psychology and Language Sciences University College London London UK; ^3^ ESRC Centre for Society and Mental Health King's College London London UK; ^4^ Department of Psychology and Neuroscience Duke University Durham NC USA; ^5^ Department of Psychiatry and Behavioral Sciences Duke University Durham NC USA; ^6^ Duke University Population Research Institute, Duke University Durham NC USA; ^7^ PROMENTA, Department of Psychology University of Oslo Oslo Norway; ^8^ Department of Child and Adolescent Psychiatry Institute of Psychiatry, Psychology & Neuroscience, King's College London London UK; ^9^ National and Specialist CAMHS Clinic for Trauma, Anxiety, and Depression, South London and Maudsley NHS Foundation Trust London UK

**Keywords:** Childhood trauma, maltreatment, measurement, risk detection

## Abstract

**Background:**

Prospective and retrospective measures of childhood maltreatment often identify different individuals and are differentially associated with psychopathology. This study examines psycho‐social factors that may explain discrepancies between these measures.

**Methods:**

Data were drawn from the Environmental Risk Longitudinal Twin Study, a nationally representative birth cohort of 2,232 children born in 1994–1995 across England and Wales and followed to age 18 (93% retention). Childhood maltreatment was assessed through: (a) prospective assessments from caregivers, researchers, and clinicians at ages 5–12, and (b) retrospective self‐reports at age 18 using the Childhood Trauma Questionnaire (for maltreatment occurring up to age 12). For the analyses, we focused on participants identified as maltreated from either measure (*n* = 290) and an a‐priori selected array of potential explanatory variables assessed between ages 5–18. We conducted two sets of analyses: comparing individuals with only prospectively identified maltreatment to those identified by both prospective and retrospective measures to understand why some participants did not retrospectively report or recall maltreatment; and comparing individuals with only retrospective self‐reports to those identified by both prospective and retrospective measures to understand why maltreatment had not been detected prospectively.

**Results:**

Participants in the prospective‐only group reported greater social support over the life course and lower psychopathology at age 18 compared to those identified through both prospective and retrospective measures. Individuals in the retrospective‐only group had higher socioeconomic status, higher self‐reported adult involvement at age 12, and less exposure to domestic violence compared to those identified through both prospective and retrospective measures.

**Conclusions:**

Our findings suggest that perceptions of social support and better mental health may buffer retrospective recall of childhood maltreatment in those with prospective measures. Furthermore, more positive family functioning and socioeconomic factors may hamper prospective detection of childhood maltreatment in those who retrospectively report it.

## Introduction

Child maltreatment is a key risk factor for adverse mental health outcomes (Agnew‐Blais & Danese, 2016; Li, D'Arcy, & Meng, 2016; Nanni, Uher, & Danese, 2012). Correctly identifying individuals who have experienced maltreatment is essential to map the underlying pathophysiological mechanisms and mitigate negative health consequences. However, considerable debate surrounds the methods for measuring and identifying childhood maltreatment (Danese, 2020; Kendall‐Tackett & Becker‐Blease, 2004; Widom, Raphael, & DuMont, 2004). For example, prospective measures of maltreatment (i.e., measured during childhood, often through parent reports or official records) identify a largely distinct group of individuals compared to retrospective measures (i.e., measured in adulthood, usually through self‐report) (Baldwin, Reuben, Newbury, & Danese, 2019), and retrospective measures are more strongly associated with psychopathology than prospective ones (Baldwin, Coleman, Francis, & Danese, 2024; Danese & Widom, 2020; Newbury et al., 2018; Reuben et al., 2016).

The low agreement between prospective and retrospective measures of maltreatment can be partially attributed to methodological differences between these measures (see Coleman et al., 2024, for a review of measurement issues). However, these discrepancies persist even in studies that use the same methods for both types of measures (Cohen's kappa range = 0.05–0.34 (Naicker, Norris, Mabaso, & Richter, 2017; White, Widom, & Chen, 2007)) and across studies employing a variety of prospective assessment strategies, including both informant reports and official records (Cohen's kappa range = 0.16–0.22)—suggesting that disagreement is not merely an artefact of measurement (Baldwin et al., 2019). Therefore, beyond methodological differences between these measures, additional mechanisms are also likely at play and may provide novel insights into vulnerability and resilience to maltreatment‐related psychopathology. Two important research questions emerge.

First, why do some individuals not retrospectively report maltreatment when it was identified prospectively? This may reflect limited memory encoding due to young age (Pillemer & White, 1989), or failure to appraise the experience as maltreatment – shaped by perceptions of normality (Berger, Knutson, Mehm, & Perkins, 1988), personality traits (Weinberg & Gil, 2016), or perceived social support (Smith & Pollak, 2021). In adulthood, people may reappraise experiences more positively, influenced by social support (Cohen & Wills, 1985), therapy (Holmes, Arntz, & Smucker, 2021), better mental health (Taylor & Brown, 1988), or traits like extraversion (Schmidt, Jendryczko, Zurbriggen, & Nussbeck, 2023). Emotion regulation strategies, including suppression (Levy & Anderson, 2008; Stramaccia et al., 2021), and motivational factors such as shame, mistrust, or protection of others (Coleman et al., 2024), may further inhibit retrospective reporting. Throughout this paper, ‘retrospective report’ refers to whether maltreatment is identified by retrospective measures, regardless of the measurement, memory or motivational mechanism at play, while ‘recall’ refers specifically to memory‐related processes.

Second, why is maltreatment not detected prospectively in some individuals who retrospectively report it? Official records may miss less severe or more hidden cases, while parent or informant reports may be limited by awareness or willingness to disclose (Berliner & Conte, 1990; Bottoms, Goodman, Schwartz‐Kenney, & Thomas, 2002). Children may conceal maltreatment, or lack of parental closeness may reduce detection (Jensen, Gulbrandsen, Mossige, Reichelt, & Tjersland, 2005). New retrospective reports may also emerge from adult reappraisals influenced by new perspectives or evolving cultural norms, or from negatively biased recall associated with psychopathology (Dalgleish & Werner‐Seidler, 2014), neuroticism (Mayo, 1983; Weinberg & Gil, 2016), and loneliness (Cacioppo & Hawkley, 2009; Smith & Pollak, 2021).

This study examines potential reasons for measurement disagreement using data from a longitudinal birth cohort that includes both prospective and retrospective measures. Previous research in this cohort has found low agreement between prospective and retrospective measures of maltreatment, which remains across a range of severity thresholds and maltreatment subtypes (Newbury et al., 2018). Based on the hypotheses outlined above and explored in detail in our previous research review (Coleman et al., 2024), we selected potential explanatory factors that were assessed as part of the ongoing cohort study. We conducted two sets of analyses. First, we explored factors differentiating individuals with maltreatment identified through prospective assessments but no retrospective self‐report (prospective‐only) from those identified by both prospective and retrospective measures. Second, we explored factors differentiating those with retrospective self‐reports of maltreatment but no prospective identification (retrospective‐only) from those identified by both measurement approaches. To our knowledge, this is the first study to systematically test a broad range of psychosocial predictors of discordance between prospective and retrospective measures of childhood maltreatment.

A greater understanding of factors influencing retrospective self‐reports of childhood maltreatment in the context of prospective measures may shed light on resilience or vulnerability to maltreatment‐related psychopathology (Baldwin et al., 2024). In addition, a greater understanding of factors influencing prospective detection of maltreatment in those with retrospective self‐reports may provide insights into the limitations of informant‐based measures.

## Methods

The premise and analysis plan for this project were preregistered (https://sites.duke.edu/moffittcaspiprojects/files/2023/04/Coleman_2023_Mechanisms_Maltreatment_disagreement.pdf), and deviations from the pre‐registration are outlined in (Appendix S1).

### Study cohort

Participants were members of the Environmental Risk (E‐Risk) Longitudinal Twin Study, which tracks the development of a nationally representative birth cohort of 2,232 twin children born in England and Wales in 1994–1995. The original E‐Risk sample was constructed in 1999–2000, when 1,116 families (93% of those eligible) with same‐sex 5‐year‐old twins participated in home visit assessments. This sample comprised 56% monozygotic and 44% dizygotic twin pairs; sex was evenly distributed within zygosity (49% male); 90% identified as white. Full details of the sample are reported elsewhere (Moffitt & the E‐Risk Study Team, 2002).

Follow‐up home visits were conducted when the children were aged 7 (98% participation), 10 (96%), 12 (96%), and 18 (93%). Home visits at ages 5, 7, 10, and 12 included assessments with participants and their mother or an alternative primary caretaker. The home visit at age 18 included interviews only with the participants. At age 18, 2,066 participants took part in the assessments. The average age of the participants at this time was 18.4 years (*SD* = 0.36); all interviews were conducted after their 18th birthday. There were no differences between those who did and did not take part at age 18 in terms of socioeconomic status (SES) assessed when the cohort was initially defined (χ^2^ = 0.86, *p* = .65), age‐5 IQ scores (*t* = 0.98, *p* = .33), and age‐5 internalising or externalising behaviour problems (*t* = 0.40, *p* = .69 and *t* = 0.41, *p* = .68, respectively). The sample represents the full range of socioeconomic conditions in Great Britain, as reflected in the families' distribution on a neighbourhood‐level socioeconomic index (ACORN [A Classification of Residential Neighbourhoods], developed by CACI Inc. for commercial use) (Odgers et al., 2012). E‐Risk families ACORN distribution closely matches that of households nation‐wide: 25.6% of E‐Risk families live in “wealthy achiever” neighbourhoods compared to 25.3% of households nation‐wide; 5.3% vs. 11.6% live in “urban prosperity” neighbourhoods; 29.6% vs. 26.9% live in “comfortably off” neighbourhoods; 13.4% vs. 13.9% live in “moderate means” neighbourhoods; and 26.1% vs. 20.7% live in “hard‐pressed” neighbourhoods. E‐Risk underrepresents urban prosperity neighbourhoods because such households are likely to be childless.

The Joint South London and Maudsley and the Institute of Psychiatry Research Ethics Committee approved each phase of the study. Parents gave informed consent, and participants gave assent between 5 and 12 years and then informed consent at age 18.

### Measures

The E‐Risk Study has collected rich measures of maltreatment, as detailed below. Potential explanatory factors for disagreement between prospective and retrospective measures were selected based on a comprehensive literature review (Coleman et al., 2024) and E‐Risk data availability.

### Maltreatment

#### Prospective assessment

Exposure to several types of maltreatment was assessed prospectively when the E‐Risk participants were aged 5, 7, 10, and 12 (assessment at age 5 concerned maltreatment since birth). Interviewers visited the home in pairs and were trained to detect signs of abuse or neglect. During each visit, interviewers interviewed the primary caretaker (usually the mother) using a structured interview about child harm, tested the participants, and observed the family environment for evidence of neglect using the Home Observation for Measurement of the Environment (HOME) (Bradley & Caldwell, 1977). Caretakers were asked several questions about whether either of their twins had been intentionally harmed (physically or sexually) by an adult or had contact with welfare agencies. If caretakers endorsed a question, follow‐up questions were asked. Interviewers made extensive notes on what had happened and whether the participant had been physically and/or psychologically harmed.

Comprehensive dossiers were compiled for each study member with cumulative information about exposure to physical abuse by an adult; sexual abuse; physical neglect; and emotional abuse/neglect (see Appendix S2 for full details). The dossiers consisted of reports from caretakers on maltreatment, recorded narratives of the interviews with caretakers, recorded debriefings with interviewers who had coded any indication of abuse and neglect at any of the home visits, and information from clinicians when the study team made a child‐protection referral. The dossiers were reviewed by two independent researchers and rated for the presence and severity (none/mild/severe) of each type of maltreatment. Inter‐rater agreement between the coders exceeded 85% among the maltreatment cases, and discrepantly coded cases were resolved by consensus review.

As in a previous paper (Newbury et al., 2018), this study used a dichotomised version of each type of prospectively measured maltreatment which separated maltreatment scores into none/mild (0) versus severe (1). Given the low prevalence of some specific forms of maltreatment (e.g., sexual abuse and physical neglect), we created an ‘any maltreatment’ composite by combining all forms of prospectively identified severe maltreatment. A rating of severe physical abuse, sexual abuse, physical neglect, and/or emotional abuse/neglect equated to a rating of any severe prospectively identified maltreatment. See Table S1 for the prevalence of each prospectively identified maltreatment subtype.

#### Retrospective self‐reports

Maltreatment was measured retrospectively using the Childhood Trauma Questionnaire (CTQ; Bernstein & Fink, 1998), when E‐Risk participants were aged 18. The CTQ is a 25‐item questionnaire used for retrospective recall of five forms of maltreatment and has high inter‐rater reliability and construct and convergent validity (Fink et al., 1995). Participants reported on their personal experiences of physical, sexual, and emotional abuse, and physical and emotional neglect for the period before they were 12 years old (i.e., the same observational period as the prospective measure). Almost all (99.5%; *N* = 2055) E‐Risk participants who took part in the age‐18 assessment completed the CTQ. This forms our analysis sample for the present study.

Maltreatment scores were dichotomised following CTQ guidelines (Bernstein & Fink, 1998) to represent none/low (0) versus moderate/severe (1) maltreatment. Because the prospective measures combined scores for emotional abuse and neglect (see above), the CTQ scores for emotional abuse and emotional neglect were also combined for the analyses, to allow a direct comparison between prospective and retrospective measures. As with the prospective measures, a rating of severe physical abuse, sexual abuse, physical neglect, and/or emotional abuse/neglect equated to a rating of any severe retrospectively reported maltreatment. See Table S1 for the prevalence of each retrospectively reported maltreatment subtype.

### Possible explanatory factors

We identified a comprehensive set of possible explanatory factors for measurement disagreement in a research review on this topic (Coleman et al., 2024), and we mapped those factors onto those available in the E‐Risk study. We identified 27 possible explanatory factors assessed between ages 5 and 18 years, which are summarised in Table 1 and detailed in (Appendix S3).

**Table 1:** Description of E‐Risk study variables examined as possible explanatory factors for maltreatment measurement disagreement

Variable	Age	Informant	Description
*Phases 5–12*			
Sex	5	Mother	1 = Male; 2 = Female
Family socio‐economic status	5	Mother	Tertiles derived from standardised composite of parental income, education, and occupation
Domestic violence exposure	5–10	Mother	CTS measuring 12 forms of physical violence (perpetration or victimisation)
Parental monitoring – knowledge subscale	12	Mother	10 items from the Monitoring and Supervision Questionnaire assessing maternal reports on knowledge of children's activities and whereabouts
Parental monitoring – knowledge subscale	12	Self	10 items from the Monitoring and Supervision Questionnaire assessing youths' reports of their parents' knowledge about their activities and whereabouts
Adult involvement	12	Self	13 items assessing presence of a supportive adult
Unsafe neighbourhood	12	Self	1 item on whether youth felt unsafe in their neighbourhood
*Phase 18: Personality*			
Agreeableness	18	Informants	Sum of 6 BFI items, averaged across two raters
Conscientiousness	18	Informants	Sum of 6 BFI items, averaged across two raters
Neuroticism	18	Informants	Sum of 6 BFI items, averaged across two raters
Extraversion	18	Informants	Sum of 6 BFI items, averaged across two raters
Openness to experience	18	Informants	Sum of 5 BFI items, averaged across two raters
*Phase 18: Social connection*			
Social support	18	Self	12 items from the Multidimensional Scale of Perceived Social Support assessing individuals' access to supportive relationships
Loneliness	18	Self	4 items from the UCLA Loneliness Scale assessing self‐reported loneliness
*Phase 18: Psychopathology*			
Major depressive disorder	18	Self	Diagnosis based on DIS interviews
Generalised anxiety disorder	18	Self	Diagnosis based on DIS interviews
Psychotic symptoms	18	Self	7 clinically verified hallucination/delusion items
PTSD (current)	18	Self	Current diagnosis based on DIS interviews
PTSD (lifetime)	18	Self	Lifetime diagnosis based on DIS interviews
PTSD avoidance & numbing	18	Self	Symptoms based on DIS interviews
PTSD reexperiencing	18	Self	Symptoms based on DIS interviews
PTSD arousal	18	Self	Symptoms based on DIS interviews
*Phase 18: Executive Function*			
RVP A Prime	18	Self	CANTAB: taps sustained attention using a signal detection measure
RVP total false errors	18	Self	CANTAB: records impulsive jumping to respond too soon
SWM strategy	18	Self	CANTAB: records trails on which a problem‐solving strategy was applied
SWM errors	18	Self	CANTAB: assesses capacity to hold information about spatial location in active memory
SSP length	18	Self	CANTAB: measures working memory
SSP length reversed	18	Self	CANTAB: measures working memory (a more difficult measure)

For full details of measures with accompanying references, see Supplementary Material. Child's age given in years. BFI, Big Five Inventory; CANTAB, Cambridge Neuropsychological Test Automated Battery; CTS, Conflict Tactics Scale; DIS, Diagnostic Interview Schedule; E‐Risk, Environmental Risk Longitudinal Twin Study; PTSD, Post‐Traumatic Stress Disorder; RVP, Rapid visual processing; SSP, spatial span; SWM, spatial working memory.

### Statistical analysis

We categorised participants based on agreement between prospective and retrospective measures of childhood maltreatment. Two dichotomous variables, ‘any prospective maltreatment’ and ‘any retrospective maltreatment’ were used to examine the overlap as in a previous publication from our team (Newbury et al., 2018). A score of 1 on ‘any prospective maltreatment’ indicated severe prospectively identified maltreatment (physical abuse, sexual abuse, emotional abuse/neglect, and/or physical neglect), while 0 indicated no severe prospectively identified maltreatment. The same scoring applied to ‘any retrospective maltreatment’. Based on the overlap between prospective and retrospective maltreatment measures, four distinct groups were identified. The ‘no maltreatment’ group included participants not identified as maltreated by either measure (scoring 0 on both ‘any prospective maltreatment’ and ‘any retrospective maltreatment’). The ‘prospective‐only’ group comprised participants identified as maltreated solely through prospective measures, without corresponding retrospective reports (i.e., scoring 1 on ‘any prospective maltreatment’ and 0 on ‘any retrospective maltreatment’). The ‘retrospective‐only’ group included individuals reporting maltreatment retrospectively with no corresponding identification through prospective measures (i.e., scoring 0 on ‘any prospective maltreatment’ and 1 on ‘any retrospective maltreatment’). The group with ‘both’ measures included participants with maltreatment consistently detected by prospective and retrospective measures (scoring 1 on both ‘any prospective maltreatment’ and ‘any retrospective maltreatment’). We focussed on participants identified as maltreated based on either prospective or retrospective measures. Therefore, participants in the ‘no maltreatment’ group were not included in the subsequent analyses.

First, we were interested in understanding why some participants prospectively identified as having experienced maltreatment did not retrospectively report maltreatment, in comparison to those consistently identified by both prospective and retrospective measures. This can help disentangle memory or motivational factors underlying the recall of maltreatment. To examine this question, we conducted a series of univariate regression models which tested which factors were associated with belonging to the prospective‐only group compared to the group identified by both measures.

Second, we were interested in understanding why some participants without prospectively identified maltreatment retrospectively reported the experience, in comparison to those consistently identified across both measures. This could help identify potential limitations of informant‐based prospective measurement or reasons retrospective recall may be inaccurate. To examine this question, we conducted univariate regression models which tested whether factors were associated with belonging to the retrospective‐only group compared to the group identified by both measures.

As the distribution of maltreatment and of some explanatory factors may differ by sex, we adjusted for sex in all analyses. As this sample comprises twins, we accounted for the non‐independence of observations using the Huber‐White variance estimator (Rogers, 1994), which provides robust standard errors adjusted for within‐cluster correlated data. This approach adjusts for the dependency between observations within twin pairs in the sample. As a sensitivity check, multiple testing corrections using the Benjamini–Hochberg procedure (for 27 tests corresponding to the 27 explanatory variables) were applied to all univariate models. We also repeated the analyses using a broader criterion for maltreatment, including any mild or severe form of maltreatment identified prospectively or retrospectively, to test whether results differed depending on the severity threshold applied – a factor that could contribute to measurement disagreement. Multivariate analyses were conducted for all significant results in the main analyses (before multiple testing corrections) to examine their independent effects.

All analyses were conducted in R (version 4.4.1) and Stata (version 18.0).

## Results

**Figure 1:**
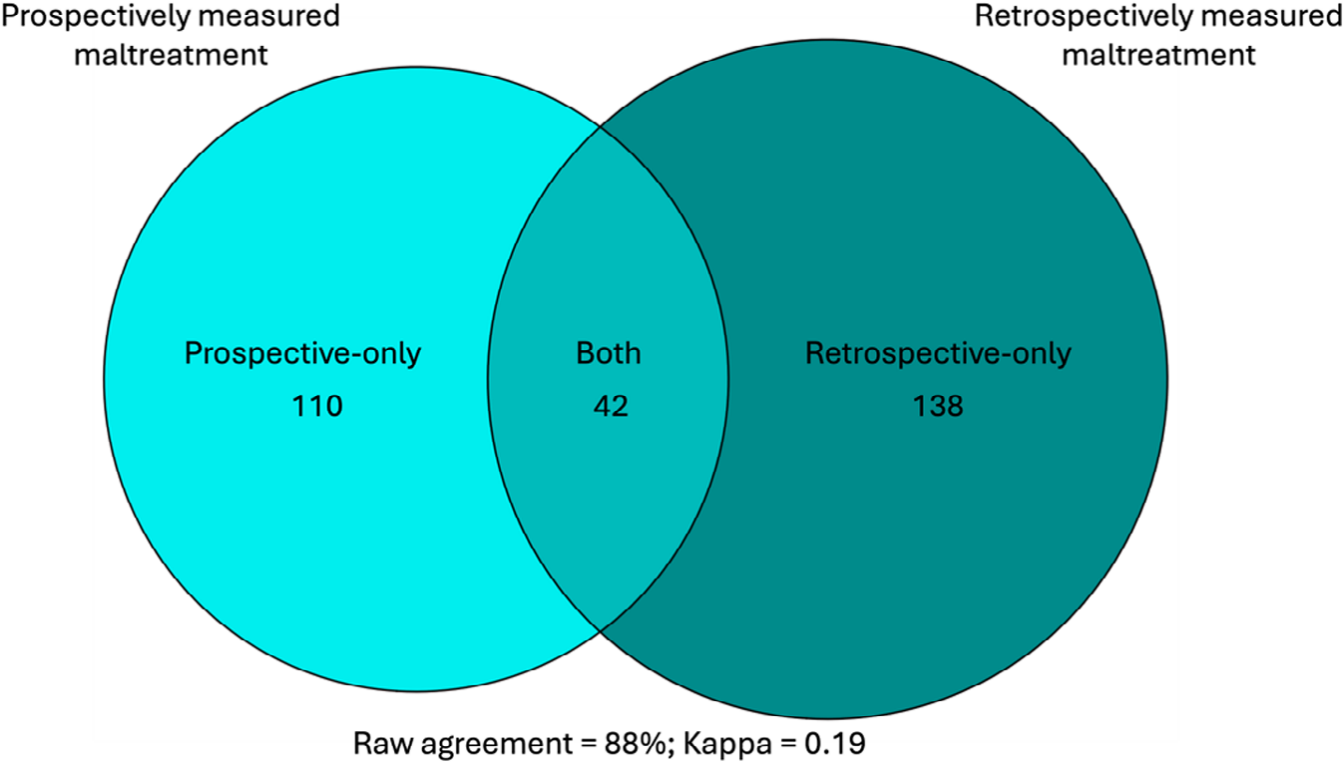
Venn Diagram showing the overlap between participants exposed to any severe maltreatment identified by prospective and retrospective measures. The Venn diagram displays the overlap between measures for all participants in the cohort identified as having experienced any severe maltreatment (*n* = 290). The remaining 1,765 participants in the cohort had no indication of maltreatment by either measure and were therefore not included. The light blue circle indicates prospectively measured maltreatment, whereas the dark blue circle indicates retrospectively reported maltreatment. The light blue non‐overlapping section (prospective‐only) shows the number of participants who were prospectively identified as experiencing maltreatment but did not retrospectively report a history of childhood maltreatment. The dark blue non‐overlapping section (retrospective‐only) shows the number of participants who retrospectively self‐reported a history of maltreatment but were not prospectively identified as experiencing maltreatment in childhood. The overlap between the two circles shows the number of maltreated participants whose exposure was identified both prospectively and retrospectively

There were 110 participants who were identified by prospective measures of maltreatment but had no retrospective report (the ‘prospective‐only’ group), 138 participants with retrospective reports of maltreatment but were not identified by prospective measures (the ‘retrospective‐only’ group), and 42 participants who were identified by both prospective and retrospective measures (the ‘both’ measures group; Figure 1). Finally, 1,765 participants had no indication of maltreatment at either time‐point and were therefore excluded from the analysis (Figure S1).

**Figure 2:**
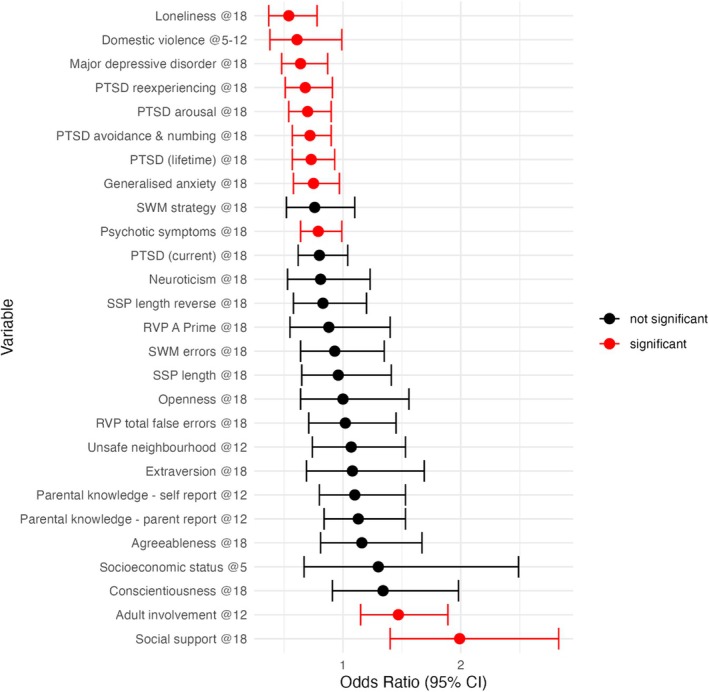
Odds ratios and confidence intervals for psychosocial variables predicting belonging to the prospective‐only maltreatment group in comparison to the group identified as maltreated by both measures (reference category). The dot indicates the point estimate (odds ratio) and the lines indicate the 95% confidence intervals. For continuous variables, odds ratios above 1 mean that higher scores on this variable predict a greater likelihood of belonging to the group with prospective‐only measures of maltreatment compared to those with consistent prospective and retrospective measures. Conversely, odds ratios below 1 mean that higher scores on this variable predict a lower likelihood of being in the prospective‐only group. For example, greater loneliness is associated with lower likelihood of belonging to the prospective‐only group, compared to those identified consistently by both prospective and retrospective measures. All models were adjusted for sex and the non‐independence of twin observations. CI, confidence intervals; PTSD, post‐traumatic stress disorder; RVP, rapid visual processing; SSP, spatial span; SWM, spatial working memory

We first tested which factors distinguished individuals with prospective‐only maltreatment from those identified by both prospective and retrospective measures (Table 2, Figure 2). During childhood, individuals in the prospective‐only group had higher adult involvement self‐reported at age 12 and lower exposure to domestic violence between ages 5–10 years. At age 18, individuals in the prospective‐only group had higher social support, were less lonely, and had lower odds of experiencing all psychiatric disorders (except current PTSD). No associations were found for factors related to personality or executive function. After multiple testing corrections, lower exposure to domestic violence, psychotic symptoms, and generalised anxiety were no longer significantly associated with the prospective‐only group. When entering all factors showing statistically significant associations before multiple testing corrections into a multivariate regression analysis, higher social support at 18 and lower exposure to domestic violence at ages 5–10 remained associated with belonging to the prospective‐only group, while higher adult involvement showed a trend towards statistical significance (total variance explained (*R* ^2^) = 25.3%) (Table 3). As a sensitivity check, we repeated the analyses including a broader criterion for maltreatment (i.e., mild or severe prospectively identified maltreatment vs. no maltreatment) (Figure S2, Tables S2 and S3). All variables that were significant in the original analysis remained significant. In addition, those in the prospective‐only group had lower odds of having a current PTSD diagnosis, were more agreeable, conscientious, and had higher scores on self‐reports of parental knowledge at age 12.

**Table 2:** Logistic regressions predicting belonging to the prospective‐only maltreatment group in comparison to the group identified as maltreated by both measures (reference category), with sample size range = 143–152

	Variable	Odds ratio [95% CI]	*p*‐Value	*p*‐Value after MTC
Phases 5–12	Socioeconomic status	1.30 [0.67, 2.49]	.436	.643
	Domestic violence	**0.61 [0.38, 0.99]**	.048	.121
	Parental knowledge – parent report	1.13 [0.84, 1.53]	.413	.643
	Parental knowledge – self‐report	1.10 [0.80, 1.53]	.546	.764
	Adult involvement	**1.47 [1.15, 1.89]**	.002	.023
	Unsafe neighbourhood	1.07 [0.74, 1.53]	.726	.861
Phase 18: Personality	Openness	1.00 [0.64, 1.56]	.993	1.000
	Conscientiousness	1.34 [0.91, 1.98]	.144	.295
	Extraversion	1.08 [0.69, 1.69]	.738	.861
	Agreeableness	1.16 [0.81, 1.67]	.427	.643
	Neuroticism	0.81 [0.53, 1.23]	.318	.566
Phase 18: Social connections	Social support	**1.99 [1.40, 2.83]**	.000	.003
	Loneliness	**0.54 [0.37, 0.78]**	.001	.016
Phase 18: Psychopathology	Major depressive disorder	**0.64 [0.48, 0.87]**	.004	.025
	Generalised anxiety	**0.75 [0.58, 0.97]**	.029	.092
	PTSD (current)	0.80 [0.62, 1.04]	.096	.223
	PTSD (lifetime)	**0.73 [0.57, 0.93]**	.009	.034
	PTSD avoidance & numbing	**0.72 [0.57, 0.90]**	.005	.027
	PTSD reexperiencing	**0.68 [0.51, 0.91]**	.008	.034
	PTSD arousal	**0.70 [0.54, 0.90]**	.007	.031
	Psychotic symptoms	**0.79 [0.64, 0.99]**	.038	.105
Phase 18: Executive function	RVP A Prime	0.88 [0.55, 1.40]	.581	.774
	RVP total false errors	1.02 [0.71, 1.45]	.933	1.000
	SWM strategy	0.76 [0.52, 1.10]	.147	.295
	SWM errors	0.93 [0.64, 1.35]	.685	.861
	SSP length	0.96 [0.65, 1.41]	.822	.920
	SSP length reverse	0.83 [0.58, 1.20]	.323	.566

Our aim was to test, within the group of participants prospectively identified as maltreated, what distinguished participants who did not retrospectively recall maltreatment from those who did. Higher odds ratios indicate that a certain characteristic was associated with greater likelihood of absent retrospective recall among those prospectively identified as maltreated. Bold text indicates significant results (at *p* < .05). All models were adjusted for sex and the non‐independence of twin observations. CI, confidence intervals; MTC, multiple testing corrections; PTSD, post‐traumatic stress disorder; RVP, rapid visual processing; SSP, spatial span; SWM, spatial working memory.

**Table 3:** Multivariate analysis predicting belonging to the prospective‐only maltreatment group in comparison to the group identified as maltreated by both measures (reference category) (*n* = 143)

Variable	Odds ratio [95% CI]	*p*‐Value
Social support @18	**1.82 [1.14, 2.91]**	.012
PTSD arousal @18	1.36 [0.75, 2.44]	.308
Adult involvement @12	1.27 [0.97, 1.67]	.079
Generalised anxiety @18	1.28 [0.90, 1.81]	.174
Major depressive disorder @18	1.13 [0.69, 1.84]	.633
Psychotic symptoms @18	1.03 [0.79, 1.35]	.814
PTSD reexperiencing @18	0.89 [0.52, 1.50]	.657
PTSD avoidance & numbing @18	0.81 [0.51, 1.30]	.387
Loneliness @18	0.69 [0.43, 1.11]	.128
PTSD (lifetime) @18	0.62 [0.32, 1.22]	.169
Domestic violence	**0.56 [0.34, 0.94]**	.029

The model was adjusted for sex and the non‐independence of twin observations. Bold text indicates significant results (at *p* < .05). CI, confidence intervals; PTSD, post‐traumatic stress disorder.

**Figure 3:**
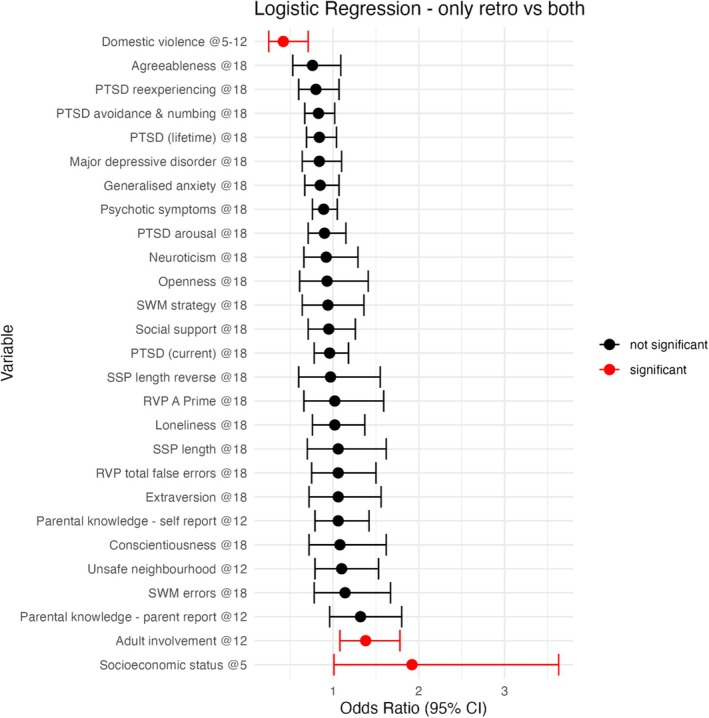
Odds ratios and confidence intervals for variables predicting belonging to the group with retrospective‐only reported maltreatment in comparison to the group identified as maltreated by both measures (reference category). The dot indicates the point estimate (odds ratio) and the lines indicate the 95% confidence intervals. For continuous variables, odds ratios above 1 mean that higher scores on this variable predict a greater likelihood of belonging to the group with retrospective‐only reports of maltreatment. Conversely, odds ratios below 1 mean that higher scores on this variable predict a greater likelihood of being in the retrospective‐only group. For example, greater adult involvement is associated with a greater likelihood of belonging to the retrospective‐only group compared to those identified consistently by both prospective and retrospective measures. All models were adjusted for sex and the non‐independence of twin observations. CI, confidence intervals; PTSD, Post‐Traumatic Stress Disorder; RVP, rapid visual processing; SWM, spatial working memory; SSP, spatial span

We then examined which factors distinguished individuals with retrospective‐only reports of maltreatment from individuals identified by both prospective and retrospective measures of maltreatment (Table 4, Figure 3). Individuals with retrospective‐only reports had higher family socioeconomic status at 5, less domestic violence in their household between ages 5–10, and higher adult involvement self‐reported at 12 years. Higher parental knowledge also showed a trend towards significance. No effects were found for factors related to psychopathology, personality, executive function, or social support. After multiple testing corrections, only lower exposure to domestic violence was associated with belonging to the retrospective‐only group. Domestic violence and adult involvement remained significant in the multivariate analysis (total variance explained (*R* ^2^) = 17.26%) (Table 5). As a sensitivity check, we repeated the analyses including a broader criterion for maltreatment (i.e., mild or severe retrospectively reported maltreatment vs. no maltreatment) (Figure S2, Tables S2 and S4). The broader severity thresholds identified a much larger retrospective‐only group (n = 545). All variables that were significant in the original analysis remained significant. In addition, those with retrospective‐only reports had lower odds of psychopathology (except generalised anxiety and current PTSD), higher scores on three executive function variables (SWM errors, SWM strategy, and SSP length), and higher scores on parent‐reported parental monitoring knowledge.

**Table 4:** Logistic regressions predicting belonging to the retrospective‐only maltreatment group in comparison to the group identified as maltreated by both measures (reference category), with sample size range = 164–180

	Variable	Odds ratio [95% CI]	*p*‐Value	*p*‐Value after MTC
Phases 5–12	Socioeconomic status	**1.92 [1.01, 3.63]**	.046	.425
	Domestic violence	**0.42 [0.25, 0.71]**	.001	.036
	Parental knowledge ‐ parent report	1.32 [0.96, 1.80]	.084	.463
	Parental knowledge – self‐report	1.06 [0.79, 1.42]	.711	.902
	Adult involvement	**1.38 [1.08, 1.78]**	.011	.158
	Unsafe neighbourhood	1.10 [0.79, 1.53]	.583	.902
Phase 18: Personality	Openness	0.93 [0.61, 1.41]	.731	.902
	Conscientiousness	1.08 [0.72, 1.62]	.703	.902
	Extraversion	1.06 [0.72, 1.56]	.757	.902
	Agreeableness	0.76 [0.53, 1.09]	.132	.463
	Neuroticism	0.92 [0.66, 1.29]	.624	.902
Phase 18: Social connections	Social support	0.95 [0.71, 1.26]	.709	.902
	Loneliness	1.02 [0.76, 1.37]	.878	.958
Phase 18: Psychopathology	Major depressive disorder	0.84 [0.64, 1.10]	.210	.534
	Generalised anxiety	0.85 [0.67, 1.07]	.169	.473
	PTSD current diagnosis	0.96 [0.78, 1.18]	.698	.902
	PTSD lifetime diagnosis	0.84 [0.69, 1.04]	.106	.463
	PTSD avoidance & numbing	0.83 [0.67, 1.02]	.074	.463
	PTSD reexperiencing	0.80 [0.60, 1.07]	.129	.463
	PTSD arousal	0.90 [0.71, 1.15]	.404	.902
	Psychotic symptoms	0.89 [0.76, 1.05]	.159	.473
Phase 18: Executive function	RVP A Prime	1.02 [0.66, 1.59]	.924	.958
	RVP total false errors	1.06 [0.75, 1.50]	.741	.902
	SWM strategy	0.94 [0.64, 1.36]	.729	.902
	SWM errors	1.14 [0.78, 1.67]	.507	.902
	SSP length	1.06 [0.70, 1.62]	.772	.902
	SSP length reverse	0.97 [0.60, 1.55]	.891	.958

Our aim was to test, within the group of participants with retrospective recall of childhood maltreatment, what distinguished those without prospective identification from those with it. Higher odds ratios indicate that a certain characteristic was associated with a greater likelihood of retrospective recall occurring in the absence of prospective identification. All models were adjusted for sex and the non‐independence of twin observations. Bold text indicates significant results (at *p* < .05). CI, confidence intervals; MTC, multiple testing corrections; PTSD, post‐traumatic stress disorder; RVP, rapid visual processing; SSP, spatial span; SWM, spatial working memory. [Corrections added on 19 February 2026, after first online publication: Odds ratio [95% CI] for Socioeconomic status, and Adult involvement were corrected from ‘1.91 [1.01, 3.63]’ to ‘1.92 [1.01, 3.63]’ and ‘0.38 [1.08, 1.78]’ to ‘1.38 [1.08, 1.78]’, respectively, and *p*‐Value for RVP total false errors was corrected from ‘741’ to ‘.741’, in this version.]

**Table 5:** Multivariate analysis predicting belonging to the retrospective‐only maltreatment group in comparison to the group identified as maltreated by both measures (reference category) (*n* = 164)

Variable	Odds ratio [95% CI]	*p*‐Value
Adult involvement @12	**1.37 [1.04, 1.82]**	.027
Socioeconomic status	1.86 [0.95, 3.64]	.068
Domestic violence @12	**0.49 [0.27, 0.87]**	.015

The model was adjusted for sex and the non‐independence of twin observations. Bold text indicates significant results (at *p* < .05). CI, confidence intervals.

## Discussion

This study tested whether a range of psycho‐social factors could distinguish between participants identified by prospective and/or retrospective measures of childhood maltreatment. The findings highlight possible factors contributing to maltreatment measure disagreement, suggesting underlying mechanisms and avenues for further research. Sensitivity analyses using broader definitions of maltreatment showed that all variables identified in the main analyses remained significant, increasing confidence that the findings are not simply artefacts of arbitrary thresholds for severity.

With regard to our first research question, we found that participants with prospective‐only maltreatment reported higher perceptions of social support across their lives and lower current psychopathology compared to those with consistent prospective and retrospective measures. Our findings suggest adaptive processes linking strong social support and better mental health with the absence of retrospective recall of maltreatment. Our findings are consistent with the results recently published from an independent cohort (Russotti et al., 2025).

Several interconnected mechanisms could account for these findings. Greater social support over the life course may directly mitigate maltreatment's impact by facilitating recognition and intervention, or indirectly by offering support that encourages cognitive reframing, allowing individuals to appraise past maltreatment in a less harmful light (Cohen & Wills, 1985; Eisenberger, 2013; Sippel, Pietrzak, Charney, Mayes, & Southwick, 2015). This buffering effect may reduce the likelihood of retrospective recall or the motivation to report. The fact that this group also had lower exposure to domestic violence may suggest a more supportive home environment, which could again buffer the impact of maltreatment over time, making it easier to appraise the experience less negatively. Additionally, positive social support and being less lonely at the time of retrospective reporting may lessen the likelihood of perceiving events as adverse, therefore lowering the chance of retrospective reporting (Smith & Pollak, 2021).

Positive social functioning and better mental health may also lead to more favourable appraisals of past negative experiences, or positively biased recall (Adler & Pansky, 2020). According to the ‘Depressive Realism Hypothesis’ and ‘Positive Illusion Theory,’ psychologically healthy individuals tend to recall the past more positively in comparison to those with poor mental health, which serves as an important affect‐regulation mechanism that preserves well‐being after difficult experiences (Alloy & Abramson, 1979; Taylor & Brown, 1988). Furthermore, the ‘fading affect bias’ suggests that, in psychologically healthy individuals, negative emotional experiences diminish more quickly than positive ones, a pattern less prevalent in those with psychopathology (Walker & Skowronski, 2009). Therefore, individuals with good mental health may report negative experiences more positively over time compared to those with psychopathology (Colman et al., 2016; Robins et al., 1985), contributing to the findings of lower psychopathology among those without retrospective recall.

These findings also support theories about the potentially adaptive function of forgetting in the context of strong social support, where suppressing or reducing unwanted memories may enhance cognitive flexibility. Cognitive flexibility theories suggest that memories incongruent with one's current social environment are more likely to be forgotten, while experiences aligned with and relevant to the environment are more likely to be retained (Ryan & Frankland, 2022). Therefore, in the context of maltreatment, it may be less adaptive for individuals in positive social environments to recall maltreatment experiences inconsistent with their present setting – possibly explaining why participants with prospective measures and stronger social support are less likely to retrospectively report the experience compared to those with weaker social support.

Forgetting may also be psychologically adaptive (Nørby, 2015), as the ability to forget or successfully inhibit recollections of negative experiences has been proposed to protect against psychological distress (Costanzi et al., 2021; Levy & Anderson, 2008). Conversely, more frequent recall of negative experiences may worsen psychiatric disorders, as cognitive models of depression and PTSD highlight how negative memories contribute to the persistence of these disorders (Beck, 2002; Ehlers & Clark, 2000). Supporting this, recent longitudinal research demonstrates that, even after accounting for current and past psychopathology, retrospective self‐reports of childhood maltreatment are linked to a greater recurrence of subsequent emotional disorders (Danese & Widom, 2023).

Previous studies have also shown that individuals who are able to successfully suppress or forget unwanted memories tend to have higher executive function, enabling them to inhibit negative memories more effectively (Levy & Anderson, 2008). This ability could, in theory, help explain the absence of retrospective recall in some individuals. However, our findings did not support this relationship. It is possible that memories of more complex types of trauma (i.e., maltreatment) may be harder to inhibit or that the tests we used to assess executive function may not have captured specific aspects of inhibitory control relevant to memory suppression.

Interestingly, in our sensitivity analyses, we also found that higher conscientiousness and agreeableness were associated with belonging to the prospective‐only group. These personality traits may promote more positive appraisals or reappraisals of maltreatment experiences, potentially contributing to the absence of retrospective reporting (Barańczuk, 2019). In addition, we found that higher self‐reported parental knowledge was associated with prospective‐only status, consistent with our other findings of greater perceived social support across the life course in this group.

With regard to our second research question, we found that participants with retrospective‐only reports of maltreatment had higher family socioeconomic status, higher self‐reported adult involvement, and lower exposure to domestic violence compared to those with consistent prospective and retrospective measures. We also observed a marginal finding of lower parent‐reported parental monitoring in the retrospective‐only group, and in the sensitivity analysis using broader definitions of maltreatment, this variable and self‐reported parental monitoring showed significant associations. These results suggest that certain environmental conditions during childhood may reduce the likelihood of maltreatment detection during prospective assessments.

For instance, the presence of domestic violence may heighten research workers' vigilance to detect and report maltreatment during prospective home visits, while the absence of domestic violence may result in less attention being paid to other potential signs of maltreatment. This is consistent with research with Child Protective Services (CPS) workers, which found that the presence of domestic violence increased the likelihood of beliefs that children were being abused and should be reported to CPS (Postmus & Merritt, 2010). Similarly, low levels of adult involvement and parental monitoring may signal potential concerns to researchers and clinicians, whereas the absence of these signals could reduce the likelihood that maltreatment will be detected. Socioeconomic status (SES) may also affect detection accuracy, as studies using vignettes have shown that healthcare professionals tend to report abuse in low‐SES families more frequently than abuse in high‐SES families (Lane & Dubowitz, 2007; Laskey et al., 2012). This pattern of results is consistent with the notion that detection bias might lead to greater prospective detection of childhood maltreatment in families with other psychosocial risks (Widom, Czaja, & DuMont, 2015).

Our findings do not support the role of negatively biased appraisals or recall as an explanation for why some individuals report maltreatment retrospectively in the absence of prospective identification (Goltermann et al., 2023). We found no evidence that psychopathology, neuroticism, or loneliness – all associated with negative attentional and recall bias in prior research – differentiated the retrospective‐only group from those with consistent evidence across both measures. This suggests that these individuals were not recalling their experiences more negatively than they were experienced at the time, but rather that the prospective measures failed to capture the experiences of maltreatment. This further supports a causal explanation for the stronger association between retrospective measures of maltreatment and poor mental health (Danese & Widom, 2023), indicating that the relationship is not artificially inflated by recall bias.

In our sensitivity analysis, where the definition of maltreatment was broadened to include milder forms of maltreatment, we identified a much larger retrospective‐only group (*n* = 545). All predictors from the main analysis remained significant, increasing confidence in the robustness of the findings. However, with this lower threshold, individuals in the retrospective‐only group also showed better overall functioning than those with consistent maltreatment measures, including lower levels of psychopathology, better cognitive functioning, and greater conscientiousness.

This pattern suggests that some participants might be recalling or appraising milder or less impactful experiences as maltreatment. These findings highlight how measurement thresholds influence both the composition and interpretation of retrospectively identified groups, supporting the use of the stricter CTQ criteria recommended by Bernstein and Fink (1998), to more accurately identify individuals at elevated risk of psychopathology.

This study should be interpreted in the context of several strengths and limitations. One major strength is the use of a large, population representative cohort, which ensures that the prevalence and distribution of maltreatment reflects UK national estimates (Radford, Corral, Bradley, & Fisher, 2013). However, the effective sample size for the analyses was comparatively small (*n* = 290) as the analyses were restricted to the subset of participants who had either prospective or retrospective measures of childhood maltreatment. As such, we were able to identify explanatory factors with comparatively large (and potentially more clinically meaningful) effect sizes, but we might have overlooked other factors with smaller effect sizes because of limited statistical power. The sample size also limited our ability to explore factors contributing to measurement disagreement within specific maltreatment subtypes. This is an important area for future research, as different mechanisms may underlie disagreement depending on maltreatment type – for example, certain forms of abuse (e.g., physical abuse) may be more visible and thus more likely to be prospectively identified, whereas others (e.g., emotional abuse or neglect) may be more subjective or covert, and thus more reliant on retrospective appraisal and disclosure. Also, the study's reliance on a twin sample may limit its generalisability, although the prevalence of childhood maltreatment in the sample aligns with estimates from a similar time period in the UK general population (Radford et al., 2013), and so do the levels of agreement between prospective and retrospective measures of maltreatment (Baldwin et al., 2019). While prevalence rates identified through prospective measures were low, this is consistent with national estimates based on official records (Gilbert et al., 2009). Another strength was the comprehensive range of potential explanatory factors measured throughout childhood and at age 18, encompassing individual, family, and wider socio‐economic factors. However, several relevant factors identified in our narrative review (Coleman et al., 2024) were not available in the E‐Risk cohort, and therefore future studies are needed to test additional explanations. Retrospective measures of maltreatment and some of the factors examined were also assessed at the same time (age 18), and the directions of such associations observed are therefore unclear.

Finally, the E‐Risk Study was not specifically designed to disentangle mechanisms of disagreement between prospective and retrospective measures of maltreatment. As such, low agreement might have also emerged from differences in measurement (e.g., different informants) as well as other mechanisms tested here (Coleman et al., 2024), although this explanation alone could not have accounted for the observed disagreement (Baldwin et al., 2019; Naicker et al., 2017; White et al., 2007). In addition, several explanatory factors examined here may be best interpreted as indicators, rather than direct measures, of underlying mechanisms. Future research can build on findings from this and other studies in the area (Reuben et al., 2016; Russotti et al., 2025) to more explicitly test mechanisms of maltreatment measure disagreement. For example, such studies can be designed to minimise measurement differences (e.g., definition, informant, reporting period) in prospective and retrospective measures, and to enable careful sensitivity analyses with multiple maltreatment measures in the same people. In addition, future studies should include more direct measures of hypothesised mechanisms, such as memory processes, appraisal styles, disclosure decisions, and informant awareness – rather than relying on indirect indicators. Of course, such studies will require substantial resources, long‐term planning, and extended follow‐up. In the meantime, rigorous cohorts like the E‐Risk Study remain uniquely placed to advance the understanding of how maltreatment is identified, reported, and remembered.

## Conclusion

Our findings suggest that perceptions of social support and connectedness may contribute to the absence of retrospective recall of maltreatment among those identified as maltreated by prospective measures. This lack of recall is associated with better mental health outcomes compared to those who do recall maltreatment (Baldwin et al., 2024). Therefore, psychosocial interventions that enhance perceived social support after maltreatment might help buffer its impact on memory and mental health (Smith & Pollak, 2021). Understanding these relationships may clarify and reduce the links between subjective recall of maltreatment and psychopathology (Baldwin et al., 2024; Danese & Widom, 2023).

Additionally, the absence of prospective identification in participants who retrospectively reported maltreatment was not explained by factors associated with recall bias (e.g., psychopathology, neuroticism, or loneliness), but rather by factors affecting prospective detection, such as family dynamics and socioeconomic status. Further research is needed to better understand sources of detection bias that may contribute to measurement disagreement and serve as barriers to the identification of individuals in need of support.

## Ethical considerations

The Joint South London and Maudsley and the Institute of Psychiatry Research Ethics Committee approved each phase of the study (approval reference number – 1997/22). Parents gave informed consent, and participants gave assent between 5 and 12 years and then informed consent at age 18. The most recent ethics approval for the phase 18 data collection was granted on 4th July 2013.

**Key points**

**What's known?**

- Prospective and retrospective measures of maltreatment identify different groups, and retrospective measures are linked to higher risk for psychopathology.

**What's new?**

- We examined a comprehensive range of psychosocial factors to explain measurement disagreement.
- Participants with prospective‐only maltreatment had higher social support across their lives and lower psychopathology compared to those identified by both prospective and retrospective measures.
- Those with retrospective‐only reported maltreatment had higher family socioeconomic status, more self‐reported adult involvement at age 12, and lower exposure to domestic violence compared to those identified by both measures.

**What's relevant?**

- These findings enhance our understanding of the mechanisms underlying maltreatment measurement disagreement, highlighting factors that may influence risk or resilience for trauma‐related psychopathology and identifying potential targets for intervention.

## Supporting information


**Appendix S1.** Changes to pre‐registered analysis plan.
**Appendix S2.** Prospective assessment of maltreatment.
**Appendix S3.** E‐Risk Explanatory variables.
**Figure S1.** Study sample selection.
**Figure S2.** Venn diagram of overlap between maltreatment measures (broader definition).
**Table S1.** Prevalence by prospectively and retrospectively measured maltreatment type.
**Table S2.** Prevalence by prospectively and retrospectively measured maltreatment type (broader definition).
**Table S3.** Sensitivity analysis for prospective‐only vs. both (broader definition).
**Table S4.** Sensitivity analysis for retrospective‐only vs. both (broader definition).

## Data Availability

The data that support the findings of this study are available on request from the E‐Risk Study team. The data are not publicly available due to privacy or ethical restrictions. Requests require a concept paper describing the purpose of data access, ethics approval at the applicant's institution, and provision for secure data access (for further details, see here: https://eriskstudy.com/data‐access/).
