# Supplementary material for: Psycho‐social factors associated with disagreement between prospective and retrospective measures of childhood maltreatment
Source: J Child Psychol Psychiatry. 2026 Jan 31;67(7):1176–89. doi: 10.1111/jcpp.70129 (PMC13265631; doi:10.1111/jcpp.70129)
Supplement: Supplementary file 1 — Appendix S1. Changes to pre‐registered analysis plan. Appendix S2. Prospective assessment of maltreatment. Appendix S3. E‐Risk Explanatory variables. Figure S1. Study sample selection. Figure S2. Venn diagram of overlap between maltreatment measures (broader definition). Table S1. Prevalence by prospectively and retrospectively measured maltreatment type. Table S2. Prevalence by prospectively and retrospectively measured maltreatment type (broader definition). Table S3. Sensitivity analysis for prospective‐only vs. both (broader definition). Table S4. Sensitivity analysis for retrospective‐only vs. both (broader definition). [file JCPP-67-1176-s001.docx]

**Psycho-social factors associated with disagreement between prospective and retrospective measures of childhood maltreatment**

**Supporting Information**

**Table of Contents**

[Appendix S1: Changes to pre-registered analysis plan 2](#_Toc199408944)

[Appendix S2: Prospective assessment of maltreatment 3](#_Toc199408945)

[Appendix S3: E-Risk Explanatory variables 6](#_Toc199408946)

[Figure S1: Study sample selection 11](#_Toc199408947)

[Table S1. Prevalence by prospectively and retrospectively measured maltreatment type 12](#_Toc199408948)

[Figure S2. Venn diagram of overlap between maltreatment measures (broader definition) 13](#_Toc199408949)

[Table S2. Prevalence by prospectively and retrospectively measured maltreatment type (broader definition) 13](#_Toc199408950)

[Table S3. Sensitivity analysis for prospective-only vs. both (broader definition). 14](#_Toc199408951)

[Table S4. Sensitivity analysis for retrospective-only vs. both (broader definition). 15](#_Toc199408952)

[References 16](#_Toc199408953)

# **Appendix S1: Changes to pre-registered analysis plan**

In our original preregistered analysis plan (<https://sites.duke.edu/moffittcaspiprojects/files/2023/04/Coleman_2023_Mechanisms_Maltreatment_disagreement.pdf>), we planned to use a continuous measure of maltreatment measurement discrepancy by subtracting each participant’s prospective cumulative maltreatment score from their retrospective cumulative maltreatment score, creating a measure of directional divergence. We intended to use univariate analyses to examine the association between the continuous maltreatment discrepancy score and all relevant variables.

However, we encountered a significant issue with this approach: the majority of participants within the cohort (n=1,765) had no reports of maltreatment at either time point, resulting in a highly skewed distribution of discrepancy scores, with most participants scoring ‘0’. Since the aim of our analysis was to understand factors associated with disagreement between prospective and retrospective measures of maltreatment, including a large number of participants without a history of maltreatment would have distorted the results and possibly hampered detection of associations within the subgroup of participants with maltreatment measures.

To address this, we revised our analysis plan to focus exclusively on a subset of E-Risk Study participants identified as having experienced maltreatment (n=290). Within this subgroup, we categorised the data into three groups based on discrepancy scores: those with prospectively identified maltreatment only, those with retrospectively reported maltreatment only reports, and maltreatment identified by both measures. Although this change to the pre-registered analysis plan reduced our sample size and decreased statistical power, it ensured that our entire sample consisted of participants identified as maltreated by either prospective or retrospective measures (or both). Using these three groups, we were able to run a series of univariate logistic regression models to answer two key and focused research questions:

1. Within the group of participants identified by prospective measures of maltreatment, what distinguishes participants who do not retrospectively report maltreatment from those who do?
2. Within the group of participants who have retrospective reports of maltreatment, what distinguishes participants who are not identified by prospective measures of maltreatment from those that are?

Even within this comparatively small sample, we were able to identify explanatory factors with relatively large and potentially more clinically meaningful effect sizes, which underscore the importance of this exploratory study as a first step in understanding the mechanisms that may contribute to measurement disagreement.

# **Appendix S2: Prospective assessment of maltreatment**

We have previously reported evidence on the reliability and validity of our measurement of childhood victimisation (Danese et al*.*, 2017). Here we summarise the method. A team of interviewers visited each family at home when the twins reached ages 5, 7, 10 and 12 years. Each home-visit interview was guided by a series of questions in a booklet. Based on these interviews with the mothers, each interviewer coded in the booklet her initial impression of whether or not she thought a child had been maltreated. The interviewers also recorded notes about their experiences in the home, and if an interviewer was worried about a child, she met with the fieldwork coordinator to debrief. Sometimes, the Study had to make a referral to help a child. Codes, notes, and the fieldwork coordinator’s narratives from the debriefs have been saved over the years to create a dossier for each child with cumulative information about exposure to physical maltreatment by an adult; sexual abuse; emotional abuse and neglect; and physical neglect. All the component measures are outlined briefly below.

*Physical and sexual harm by an adult.* When the twins were aged 5, 7, 10 and 12, their mothers were interviewed about each twins’ experience of intentional harm by an adult. At age 5 we used the standardised clinical protocol from the MultiSite Child Development Project (Dodge et al., 1990; Lansford et al., 2002). At ages 7, 10, and 12 this interview was modified to expand its coverage of contexts for child harm. Interviews were designed to enhance mothers’ comfort with reporting valid child maltreatment information, while also meeting researchers’ responsibilities for referral under the U.K. Children Act. Specifically, mothers were asked whether either of their twins had been intentionally harmed (physically or sexually) by an adult or had contact with welfare agencies. If caregivers endorsed a question, research workers made extensive notes on what had happened and indicated whether physical and/or psychological harm had occurred. Under the U.K. Children Act, our responsibility was to secure intervention if maltreatment was current and ongoing. Such intervention on behalf of E-Risk families was carried out with parental cooperation in all but one case. No families left the study following intervention. Over the years of data collection, the study developed a cumulative profile for each child, comprising the caregiver reports, recorded debriefings with research workers who had coded any indication of maltreatment at any of the successive home visits, recorded narratives of the successive caregiver interviews, and information from clinicians whenever the Study team made a child-protection referral. The profiles were reviewed at the end of the age–12 phase by two clinical psychologists. Inter-rater agreement between the coders was 90% for cases for whom maltreatment was identified (100% for cases of sexual abuse), and discrepantly coded cases were resolved by consensus review. These were coded as: 0 = no physical harm at any age; 1 = probable physical harm at any age; and 2 = definite physical harm at any age.

*Emotional abuse and neglect* were coded from research workers’ narratives of the home visits at ages 5, 7, 10, and 12. We coded quite severe examples of parental behaviour observed. For example, a mother who had schizophrenia screamed and swore at the children throughout the home visit. As another example, a father who was drunk during the home visit repeatedly spoke abusively to the children in front of the research workers. We found that coders could not empirically separate emotional abuse and emotional neglect in a reliable way and thus such experiences were coded together as emotional abuse/neglect. Inter-rater agreement between the coders exceeded 85% for cases with emotional abuse and neglect, and discrepant cases were resolved by consensus review. Children with no indication of emotional abuse/neglect were coded as 0, those where there was some indication of emotionally inappropriate/potentially abusive or neglectful behaviour were coded as 1, and where there was indication of severe emotional abuse/neglect the children were coded as 2.

*Physical neglect.* The cumulative observations of the physical state of the home environment documented by the research workers during home visits to the twins at ages 5, 7, 10 and 12 were reviewed by two raters for indication of physical neglect. This was defined as any sign that the caretaker was not providing a safe, sanitary, or healthy environment for the child. This included the child not having proper clothing or food, as well as grossly unsanitary home environments. (However, this did not include a family living in a deprived or crime-ridden neighbourhood). Inter-rater agreement between the coders was 85%, and discrepantly coded cases were resolved by consensus review. Children with no indication of physical neglect were coded as 0, those for whom there was an indication of minor physical neglect were coded as 1, and where there was incidation of severe physical neglect the children were coded as 2.

# **Appendix S3: E-Risk explanatory variables**

*Phases 5-12 Variables*

*Sex*

Sex was reported by mothers at study baseline (1 = male; 2 = female).

*Socio-economic status*

Family socioeconomic status (SES) was defined at age 5 using a standardised composite of parental income (i.e., total household income), education (i.e., highest parent qualification), and occupation (i.e., highest parent occupation). These three SES indicators were highly correlated (r=0.57–0.67) and loaded significantly onto one latent factor (Trzesniewski et al., 2006). The population-wide distribution of this latent factor was then divided into tertiles (i.e., low-, medium-, and high-SES).

*Domestic violence exposure*

Mothers reported about perpetration of and victimisation involving 12 forms of physical violence (e.g., slapping, hitting, kicking, strangling) from the Conflict Tactics Scale (CTS; Straus, Hamby, Finkelhor, Moore, & Runyan 1998), on three assessment occasions during the child’s first decade of life (when children were 5, 7, and 10 years of age). Reports of either perpetration or victimisation constituted evidence of physical domestic violence. The CTS has between-partner inter-rater reliabilities of 0.76 for perpetration and 0.82 for victimisation (Magdol, Moffitt, Caspi, & Silva,1998). Families in which no physical violence took place were coded as 0 (55.2%); families in which physical violence took place on one occasion were coded as 1 (28.0%); and families in which physical violence took place on multiple occasions were coded as 2 (16.8%).

*Parental monitoring - knowledge subscale (mother and self-report)*

Mothers’ reports on the knowledge of their children’s activities and whereabouts were collected at age 12 using 10 items from the Monitoring and Supervision Questionnaire (Stattin & Kerr, 2000). Mothers were asked about their knowledge during the last 6 months. Mothers reported on whether they knew the friends their child hangs out with, where they go in their spare time, how they spend their money, what type of homework or tests and projects they have, and how their child performs in different subjects. Answers were recorded as no, never (0), sometimes (1), and yes, always (2).

Youths’ reports of their parents’ knowledge were also collected when they were 12 years old, using the same items used with mothers but worded slightly differently (e.g., “Do your parents know . . .”).

*Adult involvement*

The presence of a supportive adult was assessed at age 12, when children were asked questions about whether they had a stable adult figure to rely on for basic needs and support (e.g., “There is an adult who I can tell almost anything to”; “There is an adult who I can go to if I am in trouble”). These were coded on a three-point scale (0 = ‘not true’ to 2 = ‘definitely true’). We derived a total score by summing responses to 13 items (internal consistency [α] = .85; M = 23.78; SD = 3.44). It should be noted that these questions did not ask the child to specify who the adult was, and thus, this could have been someone within or outside of their family.

*Unsafe neighbourhood*

This was assessed at age 12 when children were asked directly whether they “felt unsafe in their neighbourhood”. Answers were recorded as no (0) or yes (1).

*Phase 18 variables*

*Personality*

At age 18, participants nominated two people “who knew them well”. These informants were provided with questionnaires and asked to describe each participant using a 25-item version of the Big Five Inventory measuring the personality traits of Agreeableness, Conscientiousness, Neuroticism, Extraversion, and Openness to Experience. The majority of informant reports were provided by parents and co-twins. 2,050 participants had personality data, of whom 82.6%-82.7% had data from two co-informants (sample sizes varied slightly across the traits). Where two informants provided data, scores were averaged. Where one informant provided data, the participant's score was taken from that informant. Prior to averaging across co-informants, scores were standardised to have a mean of 0 and a standard deviation of 1. Final composite scores were then re-standardised.

*Social support*

Social support was assessed using the Multidimensional Scale of Perceived Social Support (MSPSS) which assesses individuals’ access to supportive relationships with family, friends and significant others (Zimet, Dahlem, Zimet, & Farley 1988). The 12 items in the MSPSS consist of statements such as ‘There is a special person who is around when I am in need’ and ‘I can count on my friends when things go wrong’. At age 18, participants rated these statements as ‘not true’ (0), ‘somewhat true’ (1), or ‘very true’ (2). We summed the scores to produce an overall social support scale with higher scores reflecting greater social support (internal consistency: α = 0.88)

*Loneliness*

Self-reported loneliness was assessed when participants were 18 years of age using 4 items from the University of California, Los Angeles (UCLA) Loneliness Scale, Version 3 (Russell, 1996): “How often do you feel that you lack companionship?”, “How often do you feel left out?”, “How often do you feel isolated from others?”, and “How often do you feel alone?”. The scale was administered as part of a computer-based self-complete questionnaire. The items were rated hardly ever (0), some of the time (1), or often (2). Items were summed to produce a total loneliness score.

*Psychopathology*

Private face-to-face interviews were conducted with participants at age 18 using the Diagnostic Interview Schedule (DIS; Robins et al., 1995). Mental health measures included the presence of major depressive disorder, generalised anxiety disorder, psychotic symptoms, current and lifetime posttraumatic stress disorder (PTSD), and symptoms of PTSD (increased arousal, reexperiencing, or avoidance or numbing).

*Executive function*

At age 18 years, participants underwent neuropsychological testing. The CANTAB (Cambridge Neuropsychological Test Automated Battery; www.cantab.com; CANTAB Eclipse Test Administration Guide, 2006, Cambridge Cognition, Cambridge, UK) (Sahakian & Owen, 1992) was administered using a touchscreen tablet computer during home visits. These measures are modestly correlated with each other (between .17 and .44), and modestly correlated with the WAIS-IV IQ (between .22 and .45). Reliability information is provided by Cambridge Cognition.

We used three CANTAB tests that tap executive functions: Rapid Visual Processing (RVP), Spatial Working Memory (SWM), Spatial Span (SSP).

1. Rapid Visual Processing: A’ (A-prime), a signal-detection measure that taps sustained attention, often called attentional vigilance. The participant scans for a 3-digit target sequence in a digit stream that is ongoing for 7 minutes, and responds whenever a target sequence is spotted. At the most difficult level, the participant scans simultaneously for two target sequences. Higher scores are better; Test-retest reliability=.76;
2. Rapid Visual Processing: Total False Alarms records impulsive jumping to respond too soon before the correct target digit sequence is complete. Because relatively few participants made numerous false alarms, this measure is categorical, coded 0=none, 1=1 false alarm, 2=2 or more false alarms; Scores were reversed so that higher scores were better, to align with the other cognitive variables. Test-retest reliability not available;
3. Spatial Working Memory: Total Errors assesses capacity to hold information about spatial location in active memory while searching for information. At the most difficult level, participants memorise 10 locations in one problem. Scores were reversed so that higher scores were better, to align with the other cognitive variables; Test-retest reliability=.70;
4. Spatial Working Memory: Strategy records trials on which the participant applied a problem-solving strategy by opening boxes in a systematic sequence. Scores were reversed so that higher scores were better, to align with the other cognitive variables; Test-retest reliability=.63;
5. Spatial Span is the visual non-verbal equivalent of the oral-auditory test Digit Span forward, and measures working memory. At the most difficult level, participants memorise a sequence of 9 coloured stimuli. Higher scores are better; Test-retest reliability=.64;
6. Spatial Span Reversed is the visual non-verbal equivalent of the oral-auditory test Digit Span backward, and is a more difficult measure of working memory. Higher scores are better. Test-retest reliability not available.

# **Figure S1: Study sample selection**

**
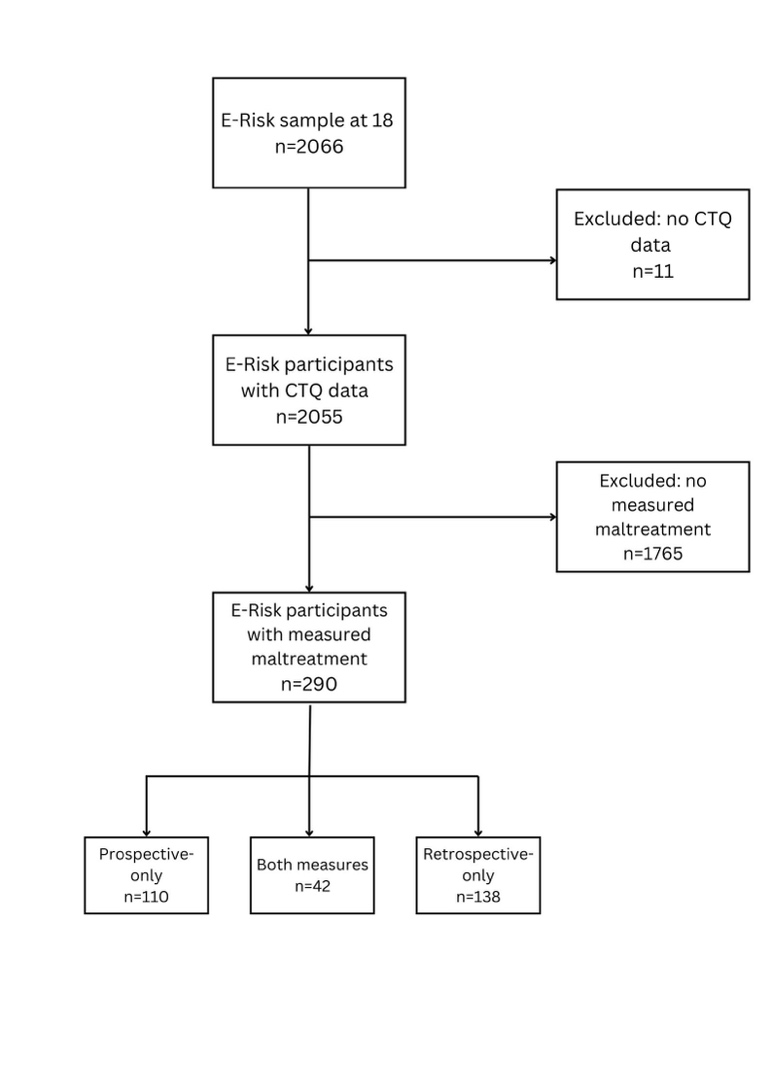
**

*Abbreviations.* CTQ, Childhood Trauma Questionnaire. E-Risk, Environmental Risk Longitudinal Twin Study.

# **Table S1. Prevalence by prospectively and retrospectively measured maltreatment type**

| Maltreatment type | Prevalence of maltreatment | | |
| --- | --- | --- | --- |
|  | Prospective  n (%) | Retrospective  n (%) | Concordance  n (%) |
| Physical abuse | 107 (5.2) | 52 (2.5) | 18 (0.8) |
| Sexual abuse | 15 (0.7) | 23 (1.1) | 6 (0.2) |
| Physical neglect | 34 (1.7) | 27 (1.3) | 4 (0.2) |
| Emotional abuse/neglect | 61(3.0) | 151 (7.4) | 20 (0.9) |

*Note.* Percentages indicate the proportion of the total study sample (n=2055) identified by each each type of maltreatment measure.

# **Figure S2. Venn diagram of overlap between maltreatment measures (broader definition)**


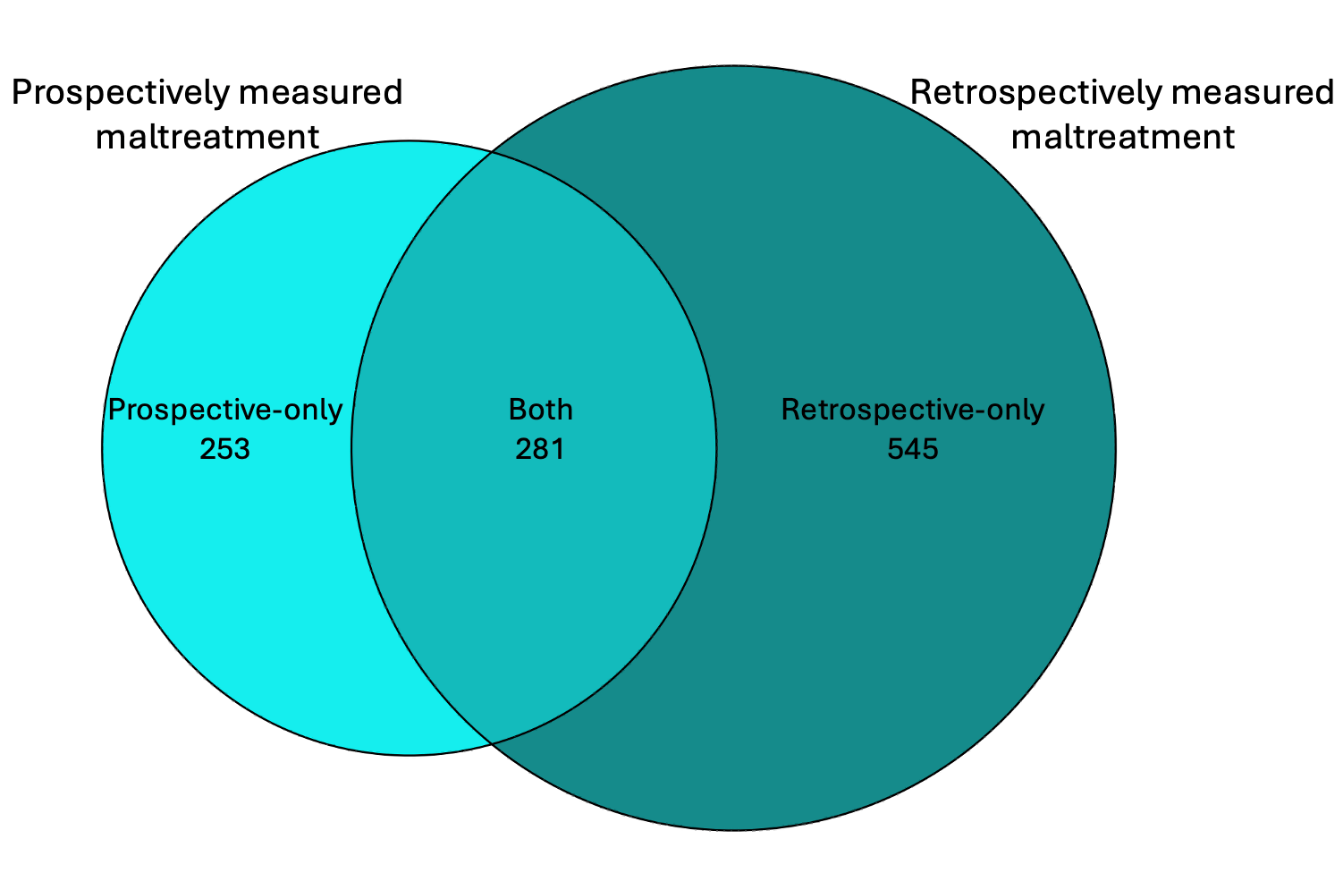


# **Table S2. Prevalence by prospectively and retrospectively measured maltreatment type (broader definition)**

| Maltreatment type | Prevalence of maltreatment | | |
| --- | --- | --- | --- |
|  | Prospective  n (%) | Retrospective  n (%) | Concordance  n (%) |
| Physical abuse | 409 (19.9) | 179 (8.7) | 80 (3.8) |
| Sexual abuse | 32 (1.5) | 30 (1.5) | 7 (0.3) |
| Physical neglect | 183 (8.9) | 138 (7.0) | 35 (1.7) |
| Emotional abuse/neglect | 237 (11.5) | 761 (37.0) | 131 (6.4) |

*Note.* Percentages indicate the proportion of the total study sample (n=2055) identified by each each type of maltreatment measure.

**Table S3. Sensitivity analysis for prospective-only vs. both (broader definition).** Logistic regressions predicting belonging to the prospective-only maltreatment group in comparison to the group identified as maltreated by both measures (reference category).

|  | **Variable** | **Odds Ratio [95% CI]** | **p-value** | **p-value after MTC** |
| --- | --- | --- | --- | --- |
| **Phases 5-12** | Socioeconomic status | 1.05 (0.86, 1.28) | 0.634 | 0.710 |
|  | Domestic violence | 0.88 (0.73, 1.05) | 0.155 | 0.271 |
|  | Parental knowledge - parent report | 1.06 (0.90, 1.24) | 0.484 | 0.616 |
|  | Parental knowledge - self report | **1.33 (1.13, 1.57)** | 0.001 | 0.003 |
|  | Adult involvement | **1.32 (1.10, 1.58)** | 0.003 | 0.007 |
|  | Unsafe neighbourhood | 0.91 (0.78, 1.06) | 0.228 | 0.337 |
| **Phase 18: Personality** | Openness | 0.94 (0.79, 1.13) | 0.538 | 0.627 |
|  | Conscientiousness | **1.32 (1.10, 1.57)** | 0.002 | 0.007 |
|  | Extraversion | 1.14 (0.84, 1.37) | 0.174 | 0.286 |
|  | Agreeableness | **1.41 (1.16, 1.71)** | 0.0004 | 0.002 |
|  | Neuroticism | 0.8 (0.67, 0.95) | 0.012 | 0.026 |
| **Phase 18: Social connections** | Social support | **2.48 (1.96, 3.14)** | <0.0001 | <0.0001 |
|  | Loneliness | **0.65 (0.53, 0.80)** | <0.0001 | 0.0004 |
| **Phase 18: Psychopathology** | Major depressive disorder | **0.73 (0.62, 0.86)** | 0.0002 | 0.001 |
|  | Generalised anxiety | **0.76 (0.63, 0.91)** | 0.003 | 0.007 |
|  | PTSD (current) | **0.83 (0.70, 0.98)** | 0.023 | 0.047 |
|  | PTSD (lifetime) | **0.76 (0.65, 0.89)** | 0.0008 | 0.003 |
|  | PTSD avoidance & numbing | **0.77 (0.66, 0.89)** | 0.0007 | 0.002 |
|  | PTSD reexperiencing | **0.67 (0.57, 0.80)** | <0.0001 | <0.0001 |
|  | PTSD arousal | **0.71 (0.60, 0.83)** | <0.0001 | 0.0003 |
|  | Psychotic symptoms | 0.92 (0.81, 1.06) | 0.244 | 0.342 |
| **Phase 18: Executive function** | RVP A Prime | 0.9 (0.74, 1.09) | 0.272 | 0.363 |
|  | RVP total false errors | 0.88 (0.75, 1.05) | 0.149 | 0.272 |
|  | SWM strategy | 0.98 (0.82, 1.17) | 0.815 | 0.878 |
|  | SWM errors | 0.95 (0.80, 1.12) | 0.525 | 0.627 |
|  | SSP length | 1 (0.84, 1.18) | 0.964 | 0.999 |
|  | SSP length reverse | 0.89 (0.75, 1.07) | 0.229 | 0.337 |

**Table S4. Sensitivity analysis for retrospective-only vs. both (broader definition).** Logistic regressions predicting belonging to the retrospective-only maltreatment group in comparison to the group identified as maltreated by both measures (reference category).

|  | **Variable** | **Odds Ratio [95% CI]** | **p-value** | **p-value after MTC** |
| --- | --- | --- | --- | --- |
| **Phases 5-12** | Socioeconomic status | **1.67 (1.38, 2.02)** | <0.0001 | <0.0001 |
|  | Domestic violence | **0.50 (0.42, 0.60)** | <0.0001 | <0.0001 |
|  | Parental knowledge - parent report | **1.36 (1.16, 1.58)** | <0.0001 | 0.001 |
|  | Parental knowledge - self report | **1.36 (1.17, 1.58)** | <0.0001 | 0.001 |
|  | Adult involvement | **1.26 (1.09, 1.45)** | 0.001 | 0.008 |
|  | Unsafe neighbourhood | 0.96 (0.84, 1.1) | 0.554 | 0.597 |
| **Phase 18: Personality** | Openness | 0.90 (0.77, 1.06) | 0.201 | 0.282 |
|  | Conscientiousness | **1.18 (1.02, 1.37)** | 0.027 | 0.064 |
|  | Extraversion | 0.94 (0.81, 1.09) | 0.403 | 0.483 |
|  | Agreeableness | 1.17 (1.0, 1.37) | 0.055 | 0.096 |
|  | Neuroticism | 0.92 (0.79, 1.08) | 0.301 | 0.383 |
| **Phase 18: Social connections** | Social support | 1.08 (0.94, 1.22) | 0.278 | 0.370 |
|  | Loneliness | 0.95 (0.83, 1.10) | 0.485 | 0.543 |
| **Phase 18: Psychopathology** | Major depressive disorder | **0.82 (0.71, 0.94)** | 0.004 | 0.017 |
|  | Generalised anxiety | 0.89 (0.78, 1.01) | 0.081 | 0.134 |
|  | PTSD (current) | 0.91 (0.82, 1.02) | 0.107 | 0.158 |
|  | PTSD (lifetime) | **0.86 (0.77, 0.97)** | 0.010 | 0.029 |
|  | PTSD avoidance & numbing | **0.85 (0.76, 0.96)** | 0.008 | 0.027 |
|  | PTSD reexperiencing | **0.82 (0.72, 0.94)** | 0.004 | 0.017 |
|  | PTSD arousal | **0.87 (0.77, 0.99)** | 0.031 | 0.067 |
|  | Psychotic symptoms | **0.87 (0.78, 0.98)** | 0.026 | 0.064 |
| **Phase 18: Executive function** | RVP A Prime | 1.07 (0.91, 1.26) | 0.414 | 0.483 |
|  | RVP total false errors | 0.99 (0.86, 1.15) | 0.929 | 0.963 |
|  | SWM strategy | 1.15 (0.98, 1.36) | 0.096 | 0.149 |
|  | SWM errors | **1.22 (1.05, 1.43)** | 0.011 | 0.029 |
|  | SSP length | **1.18 (1.01, 1.39)** | 0.041 | 0.078 |
|  | SSP length reverse | **1.19 (1.01, 1.41)** | 0.042 | 0.077 |

# **References**

Danese, A., Moffitt, T. E., Arseneault, L., Bleiberg, B. A., Dinardo, P. B., Gandelman, S. B., Houts, R., Ambler, A., Fisher, H. L., Poulton, R., & Caspi, A. (2017). The origins of cognitive deficits in victimized children: implications for neuroscientists and clinicians. *American Journal of Psychiatry, 174*, 349-361.

Dodge, K. A., Bates, J. E., & Pettit, G. S. (1990). Mechanisms in the cycle of violence. *Science, 250*, 1678-1683.

Lansford, J. E., Dodge, K. A., Pettit, G. S., Bates, J. E., Crozier, J., & Kaplow, J. (2002). A 12-year prospective study of the long-term effects of early child physical maltreatment on psychological, behavioral, and academic problems in adolescence. *Archives of Pediatrics and Adolescent Medicine, 156*, 824-830.

Magdol, L., Moffitt, T. E., Caspi, A., & Silva, P. A. (1998). Developmental Antecedents of Partner Abuse: A Prospective-Longitudinal Study. *Journal of Abnormal Psychology*, *107*, 375–389.

Robins, L., Cottler, L., Bucholz, K., Compton, W., North, C., & Rourke, K. (1995). *Diagnostic Interview Schedule for DSM-IV*. Washington University Press: St Louis.

Russell, D. W. (1996). UCLA Loneliness Scale (Version 3): Reliability, Validity, and Factor Structure. *Journal of Personality Assessment*, *66*, 20–40. <https://doi.org/10.1207/s15327752jpa6601_2>

Sahakian, B. J., & Owen, A. M. (1992). Computerized assessment in neuropsychiatry using CANTAB: discussion paper. *Journal of the Royal Society of Medicine*, *85*, 399–402.

Stattin, H., & Kerr, M. (2000). Parental Monitoring: A Reinterpretation. *Child Development*, *71*, 1072–1085. https://doi.org/10.1111/1467-8624.00210

Straus, M. A., Hamby, S. L., Finkelhor, D., Moore, D. W., & Runyan, D. (1998). Identification of Child Maltreatment With the Parent-Child Conflict Tactics Scales: Development and Psychometric Data for a National Sample of American Parents. *Child Abuse & Neglect*, *22*, 249–270. <https://doi.org/10.1016/S0145-2134(97)00174-9>

Trzesniewski, K. H., Moffitt, T. E., Caspi, A., Taylor, A., & Maughan, B. (2006). Revisiting the association between reading achievement and antisocial behavior: new evidence of an environmental explanation from a twin study. *Child Development, 77*, 72-88.

Zimet, G. D., Dahlem, N. W., Zimet, S. G., & Farley, G. K. (1988). The multidimensional scale of perceived social support. *Journal of Personality Assessment*, *52*, 30–41
